# Time matters: exploring horizons in cardiovascular disease risk assessment

**DOI:** 10.1093/eurjpc/zwaf073

**Published:** 2025-02-27

**Authors:** Lisa Pennells, Holly Pavey, Owen A Taylor

**Affiliations:** British Heart Foundation Cardiovascular Epidemiology Unit, Department of Public Health and Primary Care, University of Cambridge, Papworth Road, Cambridge Biomedical Campus, CB2 0BB Cambridge, UK; Victor Phillip Dahdaleh Heart and Lung Research Institute, University of Cambridge, Papworth Road, Cambridge Biomedical Campus, CB2 0BB Cambridge, UK; British Heart Foundation Cardiovascular Epidemiology Unit, Department of Public Health and Primary Care, University of Cambridge, Papworth Road, Cambridge Biomedical Campus, CB2 0BB Cambridge, UK; Victor Phillip Dahdaleh Heart and Lung Research Institute, University of Cambridge, Papworth Road, Cambridge Biomedical Campus, CB2 0BB Cambridge, UK; British Heart Foundation Cardiovascular Epidemiology Unit, Department of Public Health and Primary Care, University of Cambridge, Papworth Road, Cambridge Biomedical Campus, CB2 0BB Cambridge, UK; Victor Phillip Dahdaleh Heart and Lung Research Institute, University of Cambridge, Papworth Road, Cambridge Biomedical Campus, CB2 0BB Cambridge, UK

**This editorial refers to ‘Comparing 5-year and 10-year predicted cardiovascular disease risks in Aotearoa New Zealand: national data linkage study of 1.7 million adults’, by J. Liang *et al.,*  https://doi.org/10.1093/eurjpc/zwae361.**

Globally, various cardiovascular disease (CVD) risk scores are recommended by clinical guidelines for CVD prevention, helping guide preventive interventions.^1–3^ These scores integrate an individual’s risk factor values to estimate their risk of a CVD event over a future time horizon. The chosen horizon varies by score with common options being 5-year and 10-year risks^3–5^ or sometimes longer horizons such as 30-year or even lifetime risk (e.g. risk of a CVD event by the age of 90 years).^3,6^ However, there is no consensus on the optimal horizon for guiding treatment initiation.

In this issue of the *European Journal of Preventive Cardiology*, Liang *et al.*,^7^ compared estimated risks using 5-year and 10-year CVD scores. A key strength was using national health administrative databases covering around 86% of New Zealand’s population, making the findings directly relevant to clinical practice in this population. The analysis included 1 746 665 individuals aged 30–74 years without prior CVD, followed up from 2006 to 2018. The authors developed and validated 5-year and 10-year CVD risk models. They found that the median estimated 10-year risk was ∼2.5 times higher than the 5-year risk. Most importantly, 95% of individuals ranked in the top quintile of risk were the same for both models showing that, with an appropriate choice of risk threshold, the time horizon has little impact on the selecting individuals for intervention.

These findings are not entirely surprising. Most CVD risk models utilize risk factors whose effects on CVD remain stable over time. For example a 1 unit increase in non-HDL cholesterol has a similar relative effect on CVD risk at 1, 5, or 10 years.^8^ Consequently, the ranking of individuals by risk generally remains the same across different time horizons. Differences between 5- and 10-year models would only emerge if a risk factor had significantly different impact in the short-term compared to the long-term. This is rare for CVD risk factors, as shown by Liang *et al*.’s^7^ similar hazard ratios for both horizons.

The authors emphasize that the number of individuals eligible for treatment is dependent on the chosen treatment threshold, not the risk horizon itself.^7^ They illustrate the potential numbers of New Zealanders eligible for treatment using the 5-year and 10-year time frames with common risk thresholds (5% for 5-year and 10% for 10-year risk). Since 10-year risk estimates were around 2.5 times 5-year estimates, a simple doubling of the risk threshold led to slightly greater numbers being classed as treatment-eligible with the 10-year model. Presumably a 10-year threshold of 12.5% (i.e. 2.5 times 5%) would have balanced the numbers.

This illustration will no doubt inform screening and treatment strategies in New Zealand. Furthermore, it contributes to debates on the implications of using different timeframes for CVD risk prediction. However, there are many considerations not tackled by the authors, including the strong influence of age as a risk predictor, and the potential alternative use of age as the timescale in risk modelling. In the 5- and 10-year risk models used by Liang *et al.*, and recommended by many guidelines,^1,2,5,7^ an individual’s age is simply a risk factor in the model and the timescale is the period from measurement of risk factors. Since age is the most influential risk factor, applying a single threshold across all age groups typically results in older individuals comprising most of the treatment-eligible population. An alternative is to use age as the underlying timescale.^6^ Inclusion of age as the timescale allows the evolution of risk with age to develop in a flexible way that doesn’t require specification. This handling of age could, in theory, create more differences between short- and long-term risk estimates.

Furthermore, using age as the timescale allows flexibility for risk estimation over any time horizon for a given individual. Lifetime risk predictions (e.g. risk of a CVD event by age 90 years) are made in this way and reflect longer-term risk estimation for a younger than for an older person (e.g. a 50-year estimate for a 40-year-old, while only a 10-year estimate for an 80-year-old).^6^ This approach to assessing and communicating risk is more ‘age agnostic’ and tends to identify both younger and older individuals who have high risk factors relative to their peers. For this reason, some primary prevention guidelines have recommended the use of lifetime risk estimates in younger individuals,^1^ to identify those whose risk factors put them on a trajectory to become very high risk as they age.

Lifetime risk estimation is not the only approach that reduces the impact of current age on identification of high-risk individuals. Some guidelines recommend age-specific risk thresholds for 10-year risk, with lower thresholds defining a high-risk group in younger individuals.^1,5^ This approach, like lifetime risk, increases emphasis on identifying individuals with higher risk factors compared to similarly aged peers. Likewise, ‘Heart age’ provides another age-agnostic approach to risk communication. An individual’s heart age is the age of a person with the same risk estimate, but with ‘optimal/healthy’ risk factor values. For example, a 40-year-old individual with adverse levels of risk factors might have a heart age of 50 years, providing potentially powerful motivation for them to improve their cardiovascular health.^9^ Lifetime risk, age-specific risk thresholds, and heart age are three more ‘age agnostic’ approaches to identifying high-risk individuals than the approaches assessed by Liang *et al*.^7^ However, they are methodologically more complex to compute and present challenges when comparing predictive ability and risk classification in a quantitative way. Further micro-simulation studies would be helpful to look at potential population-wide long-term benefits of the different approaches.

The choice of risk horizon significantly influences clinical communication and patients’ risk perception, impacting shared decision-making. Goals of risk communication vary by context and recipient, such as prescribing statins, aligning risk perception with estimated risk, motivating engagement with preventive actions, or enabling informed healthcare decisions that reflect personal values. For instance, communicating 1-year risk to an 85-year-old smoker differs from discussing 15-year risk with a 25-year-old with Type II diabetes. Restricting communication to a single time horizon may limit its scope, as time horizons frame risk differently. In New Zealand, 5-year risk models guide treatment decisions,^4^ replacing the 10-year risk equations used prior to 1996. Shorter horizons (e.g. 5 years) may emphasize urgency, increasing risk perception and action but, since they have smaller absolute values, without careful interpretation they may be dismissed. Conversely, longer-term estimates, such as lifetime risk, may highlight the inevitability of conditions like CVD, reinforcing early intervention. Evidence suggests timeframes impact behavioural intentions: shorter horizons are more effective in older adults, while younger individuals respond better to longer horizons.^10^ Lifetime risk estimates can increase patients’ perceived risk and willingness to engage in interventions compared to shorter horizons. Strategies combining short- and long-term risk estimates, as endorsed by ESC^1^ and AHA,^3^ may enhance communication effectiveness by fostering both immediate and sustained medical intervention and behaviour change. Ideally, risk horizon choices should reflect patient characteristics, such as age and ethnicity, to align communication goals. Further studies on the effectiveness of different risk horizons across diverse populations would support improved risk score recommendations and clinical interventions.

In summary, the authors show that absolute risk estimates over traditional 5- and 10-year timeframes rank individuals similarly and highlight the numbers likely to be at high risk among New Zealanders. However, the study does not address the potential impact of using age as the timescale or adopting age-agnostic approaches, such as age-specific thresholds, lifetime risk, or heart age. This ongoing debate underscores the challenge of balancing short-term actionable insights with long-term preventive strategies, where the choice of risk threshold ultimately drives treatment eligibility. It also highlights the importance of understanding how different time horizons influence patient perceptions of risk, motivation, and willingness to engage in preventive measures.

## Data Availability

No new data were generated or analysed in support of this research.
